# Complete Genomic Characterization of a Pathogenic A.II Strain of *Francisella tularensis* Subspecies *tularensis*

**DOI:** 10.1371/journal.pone.0000947

**Published:** 2007-09-26

**Authors:** Stephen M. Beckstrom-Sternberg, Raymond K. Auerbach, Shubhada Godbole, John V. Pearson, James S. Beckstrom-Sternberg, Zuoming Deng, Christine Munk, Kristy Kubota, Yan Zhou, David Bruce, Jyothi Noronha, Richard H. Scheuermann, Aihui Wang, Xianying Wei, Jianjun Wang, Jicheng Hao, David M. Wagner, Thomas S. Brettin, Nancy Brown, Paul Gilna, Paul S. Keim

**Affiliations:** 1 Pathogen Genomics Division, Translational Genomics Research Institute, Phoenix, Arizona, United States of America; 2 Department of Biological Sciences, Northern Arizona University, Flagstaff, Arizona, United States of America; 3 BioHealthBase/University of Texas Southwest Medical Center, Dallas, Texas, United States of America; 4 Neurogenomics Division, Translational Genomics Research Institute, Phoenix, Arizona, United States of America; 5 BioHealthBase/Northrop Grumman Information Technology, Rockville, Maryland, United States of America; 6 Bioscience Division, Los Alamos National Laboratory, Los Alamos, New Mexico, United States of America; 7 The Joint Genome Institute, Department of Energy, Walnut Creek, California, United States of America; 8 Centers for Disease Control and Prevention, Fort Collins, Colorado, United States of America; Baylor College of Medicine, United States of America

## Abstract

*Francisella tularensis* is the causative agent of tularemia, which is a highly lethal disease from nature and potentially from a biological weapon. This species contains four recognized subspecies including the North American endemic *F. tularensis* subsp. *tularensis* (type A), whose genetic diversity is correlated with its geographic distribution including a major population subdivision referred to as A.I and A.II. The biological significance of the A.I – A.II genetic differentiation is unknown, though there are suggestive ecological and epidemiological correlations. In order to understand the differentiation at the genomic level, we have determined the complete sequence of an A.II strain (WY96-3418) and compared it to the genome of Schu S4 from the A.I population. We find that this A.II genome is 1,898,476 bp in size with 1,820 genes, 1,303 of which code for proteins. While extensive genomic variation exists between “WY96” and Schu S4, there is only one whole gene difference. This one gene difference is a hypothetical protein of unknown function. In contrast, there are numerous SNPs (3,367), small indels (1,015), IS element differences (7) and large chromosomal rearrangements (31), including both inversions and translocations. The rearrangement borders are frequently associated with IS elements, which would facilitate intragenomic recombination events. The pathogenicity island duplicated regions (DR1 and DR2) are essentially identical in WY96 but vary relative to Schu S4 at 60 nucleotide positions. Other potential virulence-associated genes (231) varied at 559 nucleotide positions, including 357 non-synonymous changes. Molecular clock estimates for the divergence time between A.I and A.II genomes for different chromosomal regions ranged from 866 to 2131 years before present. This paper is the first complete genomic characterization of a member of the A.II clade of *Francisella tularensis* subsp. *tularensis*.

## Introduction

*Francisella tularensis* is the infectious agent of tularemia (rabbit fever), and is a highly virulent human and animal bacterial pathogen with past and potential future uses as a bioweapon when spread as an aerosol or as a contaminant to food and water supplies. Along with other biological agents, *F. tularensis* was tested on human subjects during Japan's 13-year WWII-era biological warfare program in occupied Manchuria with devastating effect – those that were not killed outright by the disease were “sacrificed” for further testing [1], [2]. In addition, more than 20,000 Soviet and German soldiers may have been the victims of a deliberate release of *F. tularensis* by the Soviets during WWII. The bacterium was further developed as a weapon and stockpiled by both the United States and the former Soviet Union [3]. Symptoms of infection in humans include fever, chills, headache, diarrhea, joint pain, dry cough, progressive weakness, and finally, death [4] (http://www.bt.cdc.gov/agent/tularemia/pdf/tularemiafacts.pdf). Tularemia is apparently not transmissible from person to person [3].

Tularemia occurs naturally in many parts of the world. In North America, tularemia occurs in all geographic regions with high human incidence in the middle states of Arkansas, Oklahoma, Kansas and Missouri [5]. Scandinavia is also noted for large tularemia outbreaks, though few deaths result in this region. Risk factors include rabbit hunting because these animals are disease carriers. Ticks and deer flies have been implicated as vectors [6].

There are four recognized *F. tularensis* subspecies: *tularensis* (type A), *novicida*, *holarctica* (type B), and *mediasiatica* [6]; other subspecies have been detected from the environment but not successfully cultured [7]. *F. tularensis* subsp. *holarctica* strains occur throughout the temperate regions in the northern hemisphere while type A strains are confined almost exclusively to North America. *F. tularensis* subsp. *tularensis* is a highly virulent subspecies that is composed to two major clades – A.I and A.II [8], [9]. The A.I and A.II populations are geographically distinct with A.II resident in the mountainous western U.S. and A.I centered in the south-central U.S. It has been postulated that these two populations emanated from ice age refugia and became more widely distributed during the Holocene by human activities [6]. The geographic distributions of each are distinct with important ecological contrasts, including rabbit host subspecies and tick vectors. Recently, a retrospective epidemiological analysis was performed by the CDC [5] suggesting differential mortality between A.I and A.II strains. These data may be from a highly biased sample set, but they are indicative of higher virulence in A.I strain infections. Hence, genetic differentiation, ecological correlates and epidemiology all suggest that there are important biological differences among these *F. tularensis* subsp. *tularensis* populations.

Both type A.I and A.II strains have been present in repository collections but their large genetic differences were unknown until detailed genetic analysis based upon multi-locus variable number tandem repeat analysis (MLVA) [8], [9] was performed. Subsequently, PFGE subtyping also separated *F. tularensis* subsp. *tularensis* into the same two major clades [5]. Much of the previous research was performed on standard reference strains that fortuitously were members of one or the other clade. The Schu S4 strain is a member of the A.I population while the ATCC type strain (ATCC 6223, strain B-38) [10] is a member of the A.II clade. Because the B-38 strain is attenuated, most animal studies have been performed with the highly virulent Schu S4 strain.

In conjunction with the Joint Genome Institute (JGI) facilities in Walnut Creek, CA, and Los Alamos National Laboratory, we have sequenced and closed the complete genome of a virulent type A strain from Wyoming (WY96-3418), abbreviated “WY96”, isolated from a human finger wound in 1996. This strain has been identified as a member of the A.II clade by MLVA subtyping [8] and is available through the American Type Culture Collection's Biodefense and Emerging Infections (BEI) resource center. To gain further insight into the A.I – A.II genetic differentiation, we have characterized this genome by comparing it with a recently published A.I strain, Schu S4 [11] for single nucleotide polymorphisms (SNPs), insertions and deletions (indels), structural rearrangements, and whole gene differences. These genomic differences may provide clues to differences in distribution, transmission, and virulence in conjunction with future tularemia studies.

## Results and Discussion

### General Features

**Figure 1:**
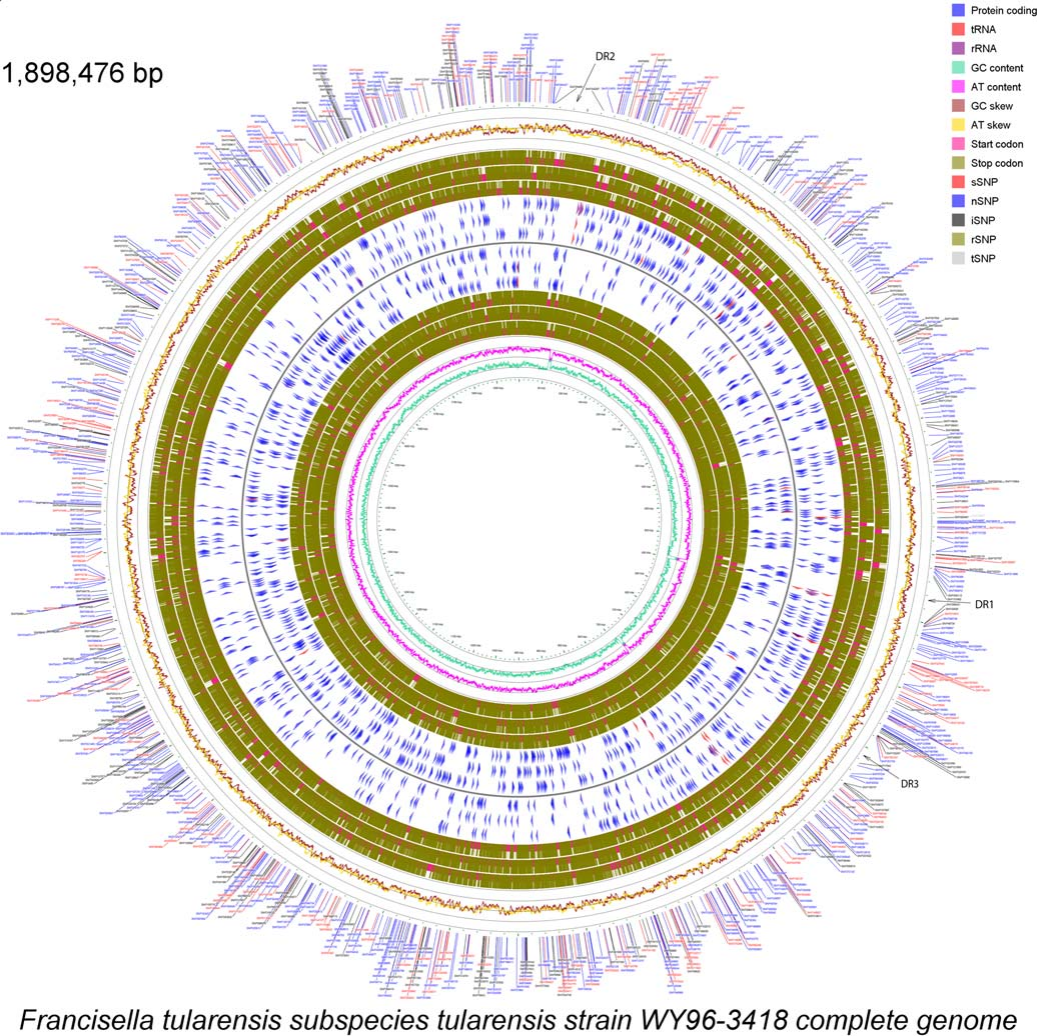
Circular genome diagram of the Francisella tularensis subsp. tularensis WY96 genome. The layers beginning with the outermost layer depict: the location and types of SNPs found between WY96 and Schu S4, a line graph depicting AT and GC skew throughout the genome, the locations of start and stop codons in the forward three reading frames, ORF locations in the forward three reading frames, ORF locations in the reverse three reading frames, start and stop codons in the reverse three reading frames, and a line graph depicting AT and GC content throughout the genome. The SNP positions are relative to the Schu S4 genome (Table S2), and are classified as synonymous (sSNP), non-synonymous (nSNP) and intergenic (iSNP), ribosomal (rSNP), and tRNA (tSNP). SNPs in duplicated regions (Table S5) are not shown here, but in Figure 4. Figure created using CGView [43].

The genomic sequence of *Francisella tularensis* subsp. *tularensis* strain WY96 has been fully annotated and deposited in GenBank (NC_009257). It consists of a single circular chromosome of size 1,898,476 base pairs (bp) derived from 38,325 shotgun reads and 8,676 finishing reads with an overall coverage of 16×. There are 1,820 open reading frames (ORFs), 52 RNA genes, and 82 transposons (Table 1, Table 2, Figure 1). In addition, there is a 33,911 bp duplication (designated DR1 and DR2) and a partial duplication of the first 5,359 bp of DR1 (designated DR3). The overall G+C content is 32.3%.

**Table 1:** General features of WY96 genome.

Length (bp)	1,898,476
GC Content (%)	32.3
Total Genes	1,820
Protein Coding Genes	1,768
Genes Assigned Function	1,303
Hypothetical Protein	282
Unclassified or Unknown Function	273
Disrupted ORFs	186
Large Duplicated regions	3
Transposons (IS elements)	82
tRNA	38
rRNA	10
Structural RNA (sRNA)	4
Average Gene Length (nt)	938
Percent Coding	90.20%

**Table 2:** IS Element Summary Table.

IS Elements	Number in WY96	Comment	Number in Schu S4
ISFtu1 (IS630 family)	52	Programmed frameshift	50
ISFtu2	19	Each with premature stop at same position compared to elements in Schu S4 genome	16
ISFtu3 (ISNCY family, ISHpal-IS1016)	5	All pseudogenes	3
ISFtu4 (IS982 family)	1	Stop codon, insertion and deletion (pseudogene)	1
ISFtu5 (IS4 family)	1	Multiple frameshifts and premature stop (pseudogene)	1
ISFtu6 (IS1595 family)	3	All pseudogenes	3
ISSod13	1	Conserved (in *Francisella tularensis*) 68 aa fragment (pseudogene) of a transposon in *Shewanella oneidensis* (364 aa)	1
Total	82		75

### Comparison to Schu S4 Genome

The WY96 genome sequence is 5,657 bp longer than Schu S4. It contains 18 regions totaling 5,208 bp of unique sequence in comparison to Schu S4, whereas Schu S4 contains 16 regions totaling 3,507 bp of unique sequence in comparison to WY96 (Table S1). WY96 contains only one gene (FTW0888 – hypothetical protein) that is not found in Schu S4, though it has 16 unique regions that overlap or are contained within genes. Schu S4, however, does not contain any unique genes, though it has 14 unique regions that overlap or are contained within genes. Comparisons of the unique regions in WY96 and Schu S4 with other *Francisella tularensis* genomes show that all of these regions are found within other subspecies genomes, so none of these regions are truly unique characteristics to these two *F. tularensis* subsp. *tularensis* genomes. WY96 has 4,382 potential polymorphisms (3,367 SNPs and 1,015 indels) in non-duplicated regions when compared to the Schu S4 genome (Table S2). There are 757 synonymous SNPs (sSNP), 1,748 non-synonymous SNPs (nSNPs), and 858 intergenic SNPs (iSNP). In non-amino-acid-coding regions, there are four SNPs in tRNA genes (tSNP) and no SNPs in rRNA genes (rSNP) (Figure 1).

### Transposable elements

Transposition activity involving the insertion and excision of insertion elements often leads to genome arrangements and mutations in the host genome. Several mechanisms such as transcriptional repressors, translational inhibitors, programmed translational frameshifting and translation termination, have been postulated for the control of transposition activity and minimization of its detrimental effects on the host cell [12].

The mechanism of programmed ribosomal frameshifting has been well characterized in the IS*911* transposon containing two consecutive, partially overlapping genes, *orfA* and *orfB*. A change in the reading frame allows translation to continue from *orfA* to *orfB,* generating a single, functional protein with the transposase activity [13]. Programmed translational frameshifting has been implicated as a control mechanism for the ISFtu1 gene in *F. tularensis* subsp. *tularensis* Schu S4 strain [11]. The full-length ISFtu1 elements in WY96 show an identical gene structure, having two consecutive ORFs and the heptanucleotide (AAAAAAG) with slippery codons allowing for potential ribosomal frameshifts.

Seven types of transposable elements were found in the WY96 genome, all of which were also found in Schu S4 (Table 2). WY96 contains 52 ISFtu1 elements, of which 47 are full-length (880–1145 bp), and five are shorter (568–578 bp). These elements contain ribosomal frameshifts and 50 copies are found in Schu S4. There are 18 ISFtu2 elements in WY96 corresponding to 16 copies in Schu S4. A unique feature of the ISFtu2 elements in WY96 is the TGG to TGA nonsense mutation at codon 68 that results in a premature stop. None of the other type A strains (Schu4 and FSC198) show a premature stop. However, a nonsense mutation from CAA to TAA at codon 20 has been reported for type B strains [14]. The biological significance of the premature stop and the possibility of a suppressor tRNA or other mechanism allowing for the low-level mis-reading of the premature stop codon remains to be investigated. The ISFtu2 elements in WY98 do not have any point mutations that lead to the loss of the original stop codon as reported for some copies of the ISFtu2 genes in the OSU18 strain [14].

There are five copies of ISFtu3 in WY96 (3 in Schu S4), two of which are intact with a single frameshift compared to Schu S4, and two contain different N-terminus truncations. There is one copy each of ISFtu4 and ISFtu5, which are intact, but contain one internal stop and 2 frameshifts. Three copies of ISFtu6 are found in WY96, all of which appear to be pseudogenes due to different truncations (N-terminus, C-terminus, and in the middle, respectively). ISSod13 is a transposon of 364 amino-acids found in *Shewanella oneidensis*. A single fragment encoding 68 amino-acids is found in WY96, which is conserved across *Francisella tularensis* species [14].

### Structural Rearrangements

**Figure 2:**
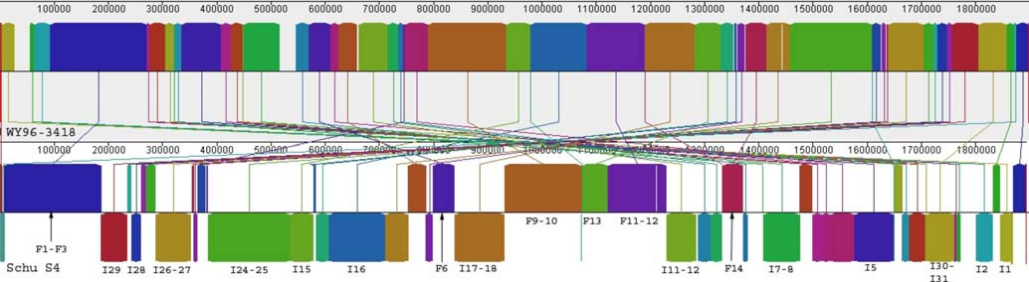
Mauve comparison of the WY96 (top) and Schu S4 (top) (bottom) genomes. Locally collinear blocks (LCBs) are the same color and size between genomes. Labeled blocks contain groups of genes. Blocks are labeled as forwards (F) and inversions (I) and the numbers correspond to names in the supplementary table (Table S3).

Several types of comparisons were made between the WY96 and Schu S4 genomes to help elucidate the structural similarities and differences between them. One comparison uses the program Mauve [15], to compare locally collinear blocks (LCBs) (Figure 2, Table S3), providing a visual overview of conserved and rearranged elements among genomes. Using a default LCB cutoff of 175, and filtering for larger blocks containing 10 Kb or larger results in 17 shared blocks (size range: 11,192–92,024 bp) in the forward direction and 31 inversions (size range: 11,133–103,724 bp). All of the LCBs in WY96 were rearranged with respect to Schu S4, and most were separated from sequential LCBs by much greater than one Kb. However, a group of three of the forward LCBs (F1–F3) was separated by only 264 bp and 188 bp, respectively. Furthermore, pairs of inversions were separated by sequences ranging from 112 bp to180 bp.

### Dot plot comparison

**Figure 3:**
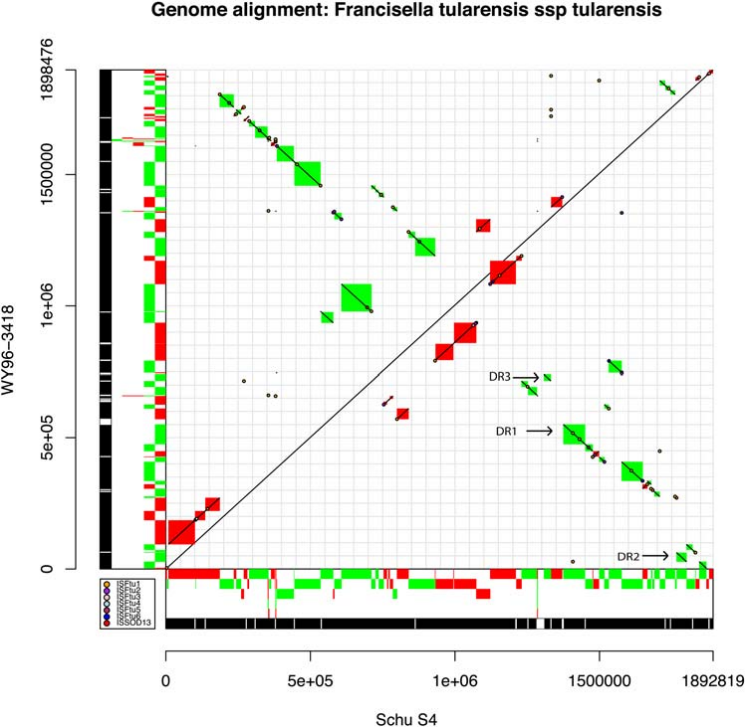
Dot plot comparison of MUMmer nucmer output [26] between Francisella tularensis subsp. tularensis strains Schu S4 and WY96. Plot generated from MUMmer coords file output using script written for R. Shared blocks of 1.5 Kb or greater were plotted as diagonal lines outlined in red (forward matches) and green (inversions). Positions of ISFtu elements (transposons) were plotted as colored spots (see legend). Red and green blocks on the axes correspond to forward and inversion matches, respectively, to the other genome, and different levels show overlapping matches. The black and white bars on each axis show overall matching regions (black) and gapped regions of no match (white). The two direct repeats (DR) diagonals are labeled DR1, DR2 along with the partial DR3 repeat (Table S4).

A dot plot comparison shows considerable rearrangement between the two genomes (Figure 3, Table S4). Among the 88 syntenic regions of 1.5 Kb or greater, 39 are forward strand matches (red), and 49 are inversions (green). All three copies of the duplicated region (DR1–DR3) are inverted between genomes. Shared ISFtu elements are mapped onto the dot plot. Of the 64 syntenic blocks of size 4 Kb or greater, 55 (86%) have an ISFtu element associated with at least one end, and 27 (42%) have an ISFtu element at both ends. Of the blocks with ISFtu elements at both ends, 60% are inverted, between Schu S4 and WY96, and 40% are not. In contrast, for blocks with ISFtu elements on only one side, 46% are inverted and 54% are not inverted.

It is not clear what biological role rearrangements play in WY96, but the genome is massively rearranged in comparison to the A.I representative, Schu S4 (Figures 2–3). Petrosino et al [14] noted a very strong correlation between transposable elements and rearrangements between the B strain, OSU18, and the A.I strain, Schu S4. We examined rearrangements between WY96 and Schu S4 in this light, and created a refined dot plot (Figure 3), which plots absolute correlated positions of all transposable elements. The correlation of ISFtu elements and rearrangements between the A.I and A.II genomes is striking, with transposable elements occurring at least one end of almost every breakpoint between syntenic regions. The IS elements ISFtu4, ISFtu5 and ISSod13 were not associated with rearrangements and are considered to be pseudogenes due to premature stop codons, deletions, or simply due to truncation (ISSod13). Two of three ISFtu6 elements and four of five ISFtu3 elements are found at rearrangement breakpoints (Figure 3), indicating that these may actually be functional, rather than pseudogenes, though they may have been inactivated after rearrangement occurred. More than half of the ISFtu2 elements are associated with rearrangements and may play an unknown functional role in rearrangement, despite the fact that they contain premature stop codons when compared to the ISFtu2 elements in Schu S4. However, it is more likely that rearrangements occur because these repeated ISFtu2 sequences serve as sites of homologous recombination. Although similar premature stop codons occur in the ISFtu2 elements in OSU18 and LVS, they are found at different positions compared to those in WY96, indicating that inactivation may have occurred independently in subspecies *holarctica* and A.II. Of the 52 ISFtu1 elements, 36 are associated with endpoints. Overall, the majority of ISFtu elements (53) are associated with rearrangements between the A.I and A.II strains, while 18 are not found at rearrangement endpoints and 10 are not involved with rearrangements at all.

### Duplicated regions

The pathogenicity island-related duplicated regions in WY96 correspond directly to those described in Schu S4, but they are shifted to different positions in the genome due to the extensive chromosomal rearrangements. Schu S4 contains a 33,911 bp duplicated region found at positions 1,374,371–1,408,281 (DR1) and 1,767,715–1,801,625 (DR2). This region contains a pathogenicity island encompassing the *IglABCD* operon. The entire region is transcriptionally regulated by *MglA*, which is implicated in virulence [16]. In WY96, DR2 is the same length as DR2 in Schu S4, though the orientation is reversed, and it is found at positions 27,170–61,080. DR1 and DR3 are likewise reversed in orientation relative to the sequences in Schu S4. DR1, found at positions 516,912–550,586, is an exact duplicate of DR2, but it is missing the first 207 bases and the last 29 bases. DR3 (which is not annotated in Schu S4, but is found in Schu S4 at positions 1,307,455–1,312,813) is found at positions 734,843–740,201 in WY96, and corresponds to the last 5,359 bases of DR1 in WY96.

**Figure 4:**
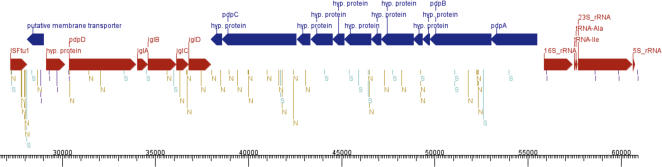
Diagram of the 33,911 bp duplicated region containing the predicted pathogenicity island. SNPs are classified as synonymous (sSNP – light blue), non-synonymous (nSNP - gold), ribosomal (rSNP – green), and intergenic (iSNP - violet). The *IglABCD* operon is shown in red. SNPs in putative and hypothetical genes are classified as intergenic because their effect is unknown (Table S5).

The WY96 DR1 and DR2 are essentially identical but share 60 polymorphisms relative to Schu S4 (Table 3, Table S5, Figure 4). DR2 contains one additional intergenic SNP found in a region missing from DR1. The Schu S4 versus WY96 DR1 and DR2 polymorphisms include: 16 synonymous SNPs, 6 conservative non-synonymous SNPs (having the same amino-acid properties), 27 non-conservative non-synonymous SNPs, 3 nonsense mutations, 1 conservative nonsense mutations (one stop codon for another), 1 “read-through mutation” (a stop codon in Schu S4 replaced with one coding for an amino-acid in WY96), three SNPs in ribosomal genes, one ribosomal gene indel, and one intergenic SNP. DR3 is a partial repeat and contains 7 ribosomal gene SNPs relative to Schu S4 (5 in 16S rDNA and 2 in 23S rDNA) and one indel in the 16S rDNA gene. Of the non-conservative SNPs, 4 were found in an ISFtu1 element, 1 in a putative major facilitator family transporter (FTW_0035 [DR1] and FTW_0532 [DR2], which is a pseudogene due to an indel-induced frameshift), 4 are found in 4 different hypothetical proteins, 2 in one hypothetical protein, 1 each in *iglB1*/*iglB2* and *iglD*1/*iglD*2, 2 in *pdpD*1/*pdpD*2 and *iglC*1/*iglC*2, 3 in *pdpB*1/*pdpB*2, and six in *pdpC*1/*pdpC*2. The *pdpC*1/*pdpC*2 gene is also interrupted by a stop codon in WY96.

The Ka/Ks ratio is one tool that may be used to quantify selection occurring between the duplicated regions. Ka is the rate of fixation for non-synonymous SNPs whereas Ks is the rate of fixation for synonymous SNPs. When Ka/Ks>1, non-synonymous SNP fixation occurs at a greater rate than synonymous SNP fixation and diversifying selection is occurring. When Ka/Ks<1, the rate of synonymous SNP fixation outpaces that of non-synonymous SNP fixation and purifying selection is occurring. Ka/Ks<1 also implies that a gene may code for a product of particular functional importance, as non-synonymous SNPs are becoming fixed at slower-than-expected rates. For this analysis, we used a cutoff of less than 1 to determine purifying selection and greater than 1 for diversifying selection.

Comparing the ratio of nSNPs to nSNP sites vs. sSNPs to sSNP sites (Ka/Ks) in the duplicated region gives a value of 0.509012, which indicates that the duplicated regions may be undergoing purifying selection, as there are more sSNPs than one would expect to find by chance [17]. Therefore, the non-synonymous substitutions are selected against, and the genes are not degrading. Ks values for the duplicated region and four comparable regions ranged from 0.0018 to 0.0043 whereas Ka values ranged from 0.0012 to 0.0016 (Table S6). The time since divergence for the duplicated region is estimated at 1,506 years whereas the time since divergence for the comparable regions ranged from 866 years to 2,131 years. All comparisons are between WY96 (A.II) and Schu S4 (A.I).

The overall Ka/Ks ratio (0.509012) observed between SchuS4 and WY96 in the pathogenicity island duplicated region (Table S6), indicates that these virulence genes are being preserved rather than degraded. On an individual basis, however, the genes in the pathogenicity island fall into several different categories, though the majority of loci are under purifying selection. In a number of cases, there are no sSNPs, and Ka/Ks is undefined, most notably for *pdpA* and *iglD*. Several loci have no nSNPs, so the Ka/Ks ratio is 0 (e.g., *iglA*). In most cases, Ka/Ks is less than 0.64, supporting the concept of purifying selection. This includes *pdpB* and *iglC*, as well as three loci that are significantly less, including *iglB*. One locus, *pdpC*, is close to neutral (1.04), and only one locus, a hypothetical protein (FTT1347), has a Ka/Ks ratio of 1.354, indicating that this locus is being degraded. Further evidence of degradation is indicated by the new stop codons generated in *pdpC*, *pdpD*, and by a conserved hypothetical protein. The biological function of duplicate genes can be happenstance due to neutral stochastic forces, for increasing transcripts for high gene expression levels (e.g., rRNA), differentiation of function (e.g., globin genes) or to protect critical genes from mutational loss. The conservation of the duplicated regions among the different *F. tularensis* subspecies argues that they have important biological function. No data currently exists to support the concept that higher transcription levels are needed or that there is differential expression of the different copies. Indeed, the highly conserved nature would argue against a development differential in transcription. What is clear is the high conservation of the genes between the WY96 repeats even in neutral codon positions. Concerted evolutionary forces may be homogenizing the sequences within the genome. Evolutionary differentiation from the Schu S4 genome is occurring, but slowly and with little effect upon the resulting amino-acid sequences. These are important genes with a critical function to the organism.

### Virulence-associated Genes

Many genes have been implicated in virulence of *Francisella tularensis* [14], [16], [18]–[21]. (Table S7, Table S8) A total of 559 SNPs are found in 231 virulence genes. Of these, 202 SNPs in 83 genes result in synonymous substitutions, 76 SNPs in 36 genes result in conservative amino-acid substitutions and 281 SNPs in 112 genes result in non-conservative amino-acid substitutions. Though conservative amino-acid substitutions may have an effect on the underlying protein structure and therefore, virulence, these latter, non-conservative changes have greater potential for affecting the virulence of WY96.

Twine et al [20] found 9 genes to be differentially expressed in an attenuated A.I strain, FSC043 when compared to the virulent A.I strain Schu S4. In particular, a 58 kD protein (FTT0918 ) was not expressed in the attenuated strain, although a fusion protein comprised of FTT0918/FTT0919 was expressed in FSC043. Petrosino et al [14] reported that FTT0918 and FTT0919 had deletions and were likewise fused in the B genome strains, LVS and OSU18. Of the 9 genes identified by Twine et al [20], all but two are altered in WY96. Notably, both FTT0918 and FTT0919 contain SNPs: FTT0918 has a non-conservative substitution, which may affect virulence, while FTT0919 contains a conservative non-synonymous substitution. Among the other members of the 9-gene group, two of these have synonymous SNPs (FTT0049 and FTT1129c) and one has only a conservative non-synonymous SNP (FTT0007); however, two of the genes (FTT0192, a lysyl-tRNA synthetase, and FTT0655, a hypothetical protein) have non-conservative amino-acid changes with a potential effect on virulence.

Type IV pili genes are implicated in virulence in *Francisella tularensis* as well as other pathogenic bacteria [18]. Of the Type IV pili genes in *F. tularensis*, 9 contain non-synonymous SNPs in WY96. Three of these (FTT0888c, FTT0889c and FTT1158c) have conservative amino-acid changes and would not be expected to have altered expression. However, the remaining 6 have non-conservative changes, and two of these (FTT0905, a Type IV pili glycosylation protein, and FTT1156c, *pilQ*) have 5 and 6 non-conservative amino-acid changes, respectively. In addition, FTT0905 is under transcriptional regulation of *MglA*, which is strongly implicated in virulence as it controls expression of the *Francisella tularensis* pathogenicity island [16]. The other Type IV pili genes with non-conservative substitutions are *pilC* (FTT1134) and *pilD* (FTT0683c), as well as two Type IV pili fiber building block proteins (FTT0861c and FTT1314c).

A total of 102 genes in *Francisella tularensis* Schu S4, LVS and U112 are regulated by *MglA* [16], which regulates the *Francisella* pathogenicity island. In WY96, 17 genes have conservative amino-acid substitutions. However, 54 genes have non-conservative SNPs. Three of the non-conservative SNPs affect stop codons, with two changing a stop to an amino-acid, and one SNP resulting in a stop codon.

Petrosino et al [14] identified 268 virulence genes associated with *Francisella tularensis*. WY96 contains 328 SNPs in 140 of these genes. One hundred twenty-five (125) synonymous SNPs are found in 88 genes and 112 genes contain a total of 204 non-synonymous SNPs. Conservative non-synonymous substitutions number 42 SNPs in 37 genes, and non-conservative substitutions total 162 in 92 genes. Of these, three genes have stop codons, two of which are in transcriptional regulators (FTT1075 and FTT1076), and two genes have nonsense mutations in Schu S4. Of the non-synonymous SNPs, 54 genes have one SNP (including, *feoB* - ferrous iron transport protein, *mr* – Ribonuclease B, and *capB* – capsule biosynthesis protein), 12 genes have two SNPs (including , *ostA*1 – organic solvent tolerance protein, *docB* – penicillin binding protein, and chitinase family 18 protein), 14 genes had 3 SNPs, and 5 genes had 4 SNPs.

*MviN* (FTW_1702) is an integral membrane protein as well as a conserved virulence factor in *Francisella tularensis* [14]. It has homology to a conserved membrane protein in a number of pathogenic bacteria, including *Coxiella burnetii, Brucella melitensis, Clostridium acetobutylicum, Mycobacterium leprae, M. tuberculosis, Neisseria meningitidis, Rickettsia conorii,* and *Yersinia pestis.* This gene has a non-conservative amino-acid substitution in WY96, significantly altering the amino-acid properties from that of proline (hydrophobic, neutral) to that of histidine (aromatic, polar, hydrophilic, charged(+)), and therefore potentially altering the structure and function of the encoded protein. The gene also has two synonymous substitutions that do not alter the characteristics of the protein.

### Virulence Attenuation

Of the large number of non-conservative amino-acid changes (281) in 112 virulence-related genes in WY96, it is likely that many of them play a role in the virulence attenuation observed in the *Francisella tularensis* A.II strains when compared to the A.I strains. Some of the non-conservative changes will have little effect, depending upon their predicted position in the resulting protein. Further informatics analyses are underway to elucidate the effects of amino-acid substitutions on the 3-dimensional structures of the proteins. Additional studies comparing these same genes in multiple taxa encompassing all subspecies of *Francisella tularensis* are also underway, and will further point to the most critical genes in determining pathogenicity.

### Divergence Rates

Five ∼39 kbp regions of the *F. tularensis* genome were compared between Schu S4 and WY96 to understand the relative divergent rates between the A.I and A.II populations. The age estimates varied from 866 to 2,131 ybp, with the duplicate regions intermediate at 1,506 ybp. The duplicated regions are not evolving at a different rate than other representative chromosomal regions. The extreme intragenomic conservation of the duplicated regions is not reflected in exceptional intergenomic conservation between the A.I and A.II genomes. An intragenomic homogenization mechanism such as gene conversion must be maintaining the two sequences but its biological importance is still undefined. The age estimates based upon molecular clock models are notoriously hard to calibrate to real time and are based upon numerous assumptions (e.g., evolutionary rate, calibration to time, constancy of rates, the neutrality of variation) that are hard to validate. Still, these estimates are younger than might be expected for the Pleistocene refugia model proposed by Farlow et al. [8].

## Materials and Methods

### Sequencing

The *Francisella tularensis* subsp. *tularensis* WY96 isolate used in the present study was obtained from the Centers for Disease Control and Prevention collection, and was isolated from a human finger wound in 1996. The genome was sequenced at the Joint Genome Institute (JGI) using a combination of 4 kb, 9 kb, and 40 kb DNA libraries. All general aspects of library construction and sequencing performed at the JGI can be found at http://www.jgi.doe.gov/. Draft assemblies were based on 41,626 total reads. The five libraries provided 16x coverage of the genome. The Phred/Phrap/Consed software package (www.phrap.com) was used for sequence assembly and quality assessment [22]–[24]. After the shotgun stage, reads were assembled with parallel phrap (High Performance Software, LLC). Possible mis-assemblies were corrected with Dupfinisher [25], transposon bombs, and PCR. Gaps between contigs were closed by editing in Consed, custom primer walk or PCR amplification (Roche Applied Science, Indianapolis, IN). The completed genome sequence of WY96 contains 41,748 reads, achieving an average of 16-fold sequence coverage per base with an error rate less than 1 in 100,000.

### Annotation

TIGR's pipeline for gene prediction (Glimmer3 [26]), automated annotation (AutoAnnotate [27] - http://manatee.sourceforge.net/) and manual curation (Manatee [27] - http://manatee.sourceforge.net/) was used for genome annotation. The first step was to re-format the sequence so that dnaA was the first gene and then reassign the sequence coordinates before submitting the sequence for gene predictions. Glimmer 3.02 was used to predict protein-coding genes using default parameters. The program tRNAscanSE [28] (version 1.23, parameters: -B) was used to find genes coding for tRNAs. A sequence similarity search using Exonerate [29] (version 1.0.0, parameters: -m NER) was used to identify 16S and 23S ribosomal RNA genes. A sequence similarity search against RFam [30] (release 7.0, rfam_scan.pl version 0.1, parameters: –bt 0.1 –nobig), a comprehensive database of non-coding RNA (ncRNA) families was used to identify genes coding for other non-coding RNAs, (such as 5S ribosomal RNAs). Prediction of ribosome binding sites (RBS) was done using RBSfinder [31] algorithm developed by TIGR (http://www.tigr.org/software/genefinding.shtml).

Gene functional predictions were performed as follows. Each predicted protein was searched against a non-redundant amino acid database (nraa) made up of all proteins available from GenBank, PIR and SWISS-PROT. The search algorithm employed for these searches is BLAST-Extend-Repraze (BER). A BLAST [32] search (blastp, e-value cutoff: 0.1) was performed for each protein against nraa and all significant matches were stored in a mini-database. Then a modified Smith-Waterman alignment [33] was performed on the protein against the mini-database of BLAST hits. In order to identify potential frameshifts or point mutations in the sequence, the gene was extended 300 nucleotides upstream and downstream of the predicted coding region. If significant homology to a match protein existed and extended into a different frame from that predicted, or extended through a stop codon, the program continued the alignment past the boundaries of the predicted coding region. All of the proteins from the genome sequences were also searched against the Pfam [34] (release 20.0) HMMs (hmmpfam HMMER version 2.3.2, parameters: -E 0.1 –cut_ga) and TIGRFAMs [35] (release 6.0) built from highly curated multiple alignments of proteins thought to share the same function or to be members of the same family. Each HMM has an associated cutoff score above which hits are known to be significant. Additional searches run on the sequence include prediction of trans-membrane helices using TMHMM [36], prediction of signal peptide with signalP [37], lipoprotein motif and COG [38] (Clusters of Orthologous Groups of proteins based on phylogenetic classification of proteins encoded in complete genomes) relationships. These data were used by AutoAnnotate to make functional predictions for proteins, which were then made available in the Manatee interface for manual evaluation of the predicted function.

### ISFtu Mapping

ISFtu mapping was done using EXONERATE [29] using the affine:bestfit model, which performed a best fit or best location alignment of the query (WY96 ISFtu elements with 0 or more bp extensions on each side of the element) onto the target sequence (Schu S4 genome). It reported alignments that included the entire query sequence. The length of the extensions determined how a query sequence was mapped. Extensions that were too short resulted in multiple mappings, and those that were too long produced no mapping. The optimal extensions varied amongst different ISFtu elements. A script was been developed to identify the optimal extension for each ISFtu element.

### SNP Discovery

SNP and indel polymorphisms between the WY96 and Schu S4 genomes were discovered using a Perl pipeline called *SIDACS* (a SNP and Indel Discovery And Classification System based on MUMmer and developed jointly between TGen and NAU) [39]. The program uses output from pairwise genomic comparisons in MUMmer [40], grouping SNPs and indels, and allowing filtering based on sequence quality metrics (phd_cutoff = 30, phd_avgcut = 30, region_all_threshhold = 20) and size of syntenic region, including flanking length (30 bp), and length to mismatch (1 bp). The SNPs were then classified as intergenic, synonymous and non-synonymous according to their positions in a coding sequence.

### Age Estimation

Four large, shared regions approximately the same size in bp as the duplicated region were identified from the MUMmer3 coords files. Sequences for each of the comparable regions along with sequence for the duplicated region were extracted from the WY96 and Schu S4 genomes and aligned using ClustalW. Aligned ORF boundaries were determined using the GenBank files as guides and confirmed by visual inspection of the alignments. dnaSP was used to calculate the number of observed and potential synonymous and non-synonymous SNPs, as well as the values for Ka and Ks for all regions [41]. Age estimates were calculated using the molecular clock rate for *E. coli* as an approximation (3.4×10^−9^ synonymous mutations/generation) and assuming 300 generations per year, as per the method employed for age estimates in *Yersinia pestis* [42].

## Supporting Information

Table S1 Unique regions in *Francisella tularensis* subsp. *tularensis* WY96 and Schu S4.
(0.03 MB XLS)

Table S2 SNPs in non-duplicated regions between *Francisella tularensis* subsp. *tularensis* Schu S4 and WY96.
(0.47 MB XLS)

Table S3 LCBs above 10 Kb between *Francisella tularensis* subsp. *tularensis* WY96 and Schu S4.
(0.03 MB XLS)

Table S4 MUMmer Coords Comparisons for Dot Plot of *Francisella tularensis* subsp. *tularensis*.
(0.04 MB XLS)

Table S5 Duplicated Region SNPs in *Francisella tularensis* subsp. *tularensis* WY96 vs Schu S4 Duplicated Regions.
(0.06 MB XLS)

Table S6 Observed and Potential Synonymous and Non-synonymous SNPs between *Francisella tularensis* subsp. *tularensis* WY96 and Schu S4 in Comparably Sized Regions.
(0.04 MB XLS)

Table S7 nSNPs and Stops in *Francisella tularensis* subsp. *tularensis* Virulence Genes.
(0.02 MB XLS)

Table S8 Non-Synonymous SNPs in Virulence Genes of *Francisella tularensis* subsp. *tularensis*.
(0.17 MB XLS)
